# Plant–Insect Interactions: Host Plant Resistance, Biological Control, and Pollination

**DOI:** 10.3390/plants14101488

**Published:** 2025-05-16

**Authors:** Francisco Rubén Badenes-Pérez

**Affiliations:** Instituto de Ciencias Agrarias, Consejo Superior de Investigaciones Científicas, 28006 Madrid, Spain; fr.badenes@csic.es

## 1. Introduction

The evolving field of plant–insect interactions impacts basic and applied fields of plant sciences, entomology, and agronomy. Within this field, pest management and plant pollination receive a lot of attention because of their implications for maximizing crop yield [1,2,3,4]. Host plant resistance and biological control are important aspects of pest management that can bring alternatives to the problems arising with insecticide use [5,6]. This Special Issue presents a collection of papers addressing the interactions of insects and mites with plants, while highlighting the importance that pest management and pollination have in this field.

## 2. Overview of Published Articles

1. Zhu et al. (Contribution 1) investigated gene expression response to mechanical damage and feeding by *Phthorimaea operculella* Zeller (Lepidoptera: Gelechiidae) at different time points. Compared to mechanical damage, feeding by *P. operculella* induced moregenes and resulted in a stronger gene expression.
2. Zhang et al. (Contribution 2) studied the adaptive responses of *S. frugiperda* Smith (Lepidoptera: Noctuidae) to nutritional and enzymatic variations in different maize cultivars. This study highlights the different adaptations of *S. frugiperda*’s digestive and detoxification systems.
3. Volp et al. (Contribution 3) investigated the avoidance of and preference for pigeonpea flowers and pods of first-, second-, third-, and fourth-instar larvae of *Helicoverpa armigera* Hübner (Lepidoptera: Noctuidae). Early instars were shown to prefer flowers, while older instars preferred to feed on pods. Boring through pigeonpea pod walls imposed a physiological cost for third- but not for fourth-instar larvae.
4. Rodríguez-Leyva et al. (Contribution 4) analyzed the volatile organic compounds (VOCs) released by the cactus pear *Opuntia ficus-indica* (L.) Miller as a result of herbivory by the cochineals *Dactylopius coccus* Costa and *D. opuntiae* Cockerell. Cactus pear produced different VOCs depending on the *Dactylopius* species affecting the plant. Among the VOCs identified, methyl salicylate, terpenes, and *p*-vinylguaiacol were suggested as being likely to play a defense role in *O. ficus-indica*.
5. Abbes et al. Al-Azzazy and Alhewairini (Contribution 5) studied the suitability of three solanaceous crops—eggplant, potato, and tomato—to cotton mealybug *Phenacoccus solenopsis* Tinsley (Hemiptera: Pseudococcidae). Using age-stage two-sex life tables, the study showed that tomato, followed by potato plants, were more suitable hosts for *P. solenopsis* than eggplant.
6. Al-Azzazy and Alhewairini (Contribution 6) tested the use of the predatory mites *Phytoseius plumifer* Canestrini and Fanzago and *Euseius scutalis* Athias-Henriot (Phytoseiidae) as biological control agents against the grape erineum mite, *Colomerus vitis* Pagenstecher (Eriophyidae).
7. George et al. (Contribution 7) investigated the potential of different fungal endophytes, specifically *Beauveria bassiana* strains, in colonizing cotton plants and their efficacy against tarnished plant bug *Lygus lineolaris* Palisot de Beauvois (Hemiptera: Miridae). These endophytes can colonize different plant parts, affecting cotton plant growth as well as the development and mortality of *L. lineolaris* adults and nymphs. The new *B. bassiana* strain JG-1 affected the olfactory response of *L. lineolaris* adults and caused significant mortality in bioassays.
8. Benvenuti (Contribution 8) reviewed the mutualistic interactions between pollinators and weeds, discussing them in terms of food reward and attractiveness, and analyzed the specialization of these interactions.
9. De Brito Machado et al. (Contribution 9) investigated how the chemodiversity of leaves and reproductive organs affect pollinator visitation in *Piper mollicomum* Kunth (Piperaceae). They showed the importance of understanding the complex interactions between plant chemistry, environmental factors, and plant–insect interactions in *P. mollicomum*.
10. Pimkornburee et al. (Contribution 10) investigated the settling preferences and feeding behavior of the silverleaf whitefly *Bemisia tabaci* Gennadius (Hemiptera: Aleyrodidae) on six cassava cultivars using electrical penetration graph techniques. They suggest that cultivars with large trichomes and a low trichome density are more resistant to whitefly infestation and the subsequent transmission of Sri Lankan cassava mosaic virus than those with smaller trichomes and a lower trichome density.
11. Jansen-González et al. (Contribution 11) investigated the parasitic relationship between *Arastichus gallicola* Ferrière (Hymenoptera: Eulophidae), an ovary-galling wasp, and the inflorescences of *Thaumatophyllum bipinnatifidum* (Schott ex Endl.) Sakur., Calazans & Mayo (Araceae). Although ovule fertilization is not required for gall formation by *A. gallicola*, pollination substantially enhanced gall retention by reducing *T. bipinnatifidum* inflorescence abscission.

## 3. Key Messages

The impact that insects have as pollinators in angiosperms is one of the most prominent aspects of studying plant–insect interactions [7]. With the decline in pollinators [8,9], their conservation is very important. Weeds can be an important part of pollinator conservation [10,11]. The process of insect galling by eulophid wasps can interact with pollination and fruit retention in trees [12,13]. Plant chemistry also plays an important role in plant–insect interactions [14]. Besides being a key part of the integrated pest management of insect pests, host plant resistance can prevent damage caused by insect-transmitted diseases [15,16]. Studies on host plant resistance and biological control are important in both generalist species that cause important economic losses in many crops, like the generalists *B. tabaci*, *H. armigera*, *L. lineolaris*, *P. solenopsis*, and *S. frugiperda* [17,18,19,20,21], and in specialists like *P. operculella*, *D. coccus*, and *D. opuntiae* that damage only a few crop species [22,23]. Endophytic entomopathogenic fungi can be used as part of the integrated pest management of some of the insect pests included in this Special Issue [24,25]. Some of these insect pests have also developed resistance to many insecticides and insecticide use presents the risk of disrupting the biological control of insect and mite pests [18,26,27,28,29,30].

## 4. Future Directions

Plant–insect interactions encompass many relationships deserving further research [31,32,33]. With climate change and habitat loss being such pressing issues [34,35], the effects that these can have on plant–insect interactions should be further studied. Studies conducted in geographical locations that are biodiversity hotspots should offer many opportunities for the study of plant–insect interactions and conservation [36]. Alternatives to insecticides should also be investigated further, especially in insect pest species that can easily develop resistance to insecticides. The compatibility of different biological control agents for improving pest management should be further investigated [37,38,39]. In terms of host–plant resistance, plants like *Barbarea vulgaris* W. T. Aiton (Brassicaceae), which can provide resistance to several types of pests, are of particular interest for further research [40,41,42,43].
